# Modeling Aβ42 Accumulation in Response to Herpes Simplex Virus 1 Infection: Two Dimensional or Three Dimensional?

**DOI:** 10.1128/JVI.02219-20

**Published:** 2021-02-10

**Authors:** Eric E. Abrahamson, Wenxiao Zheng, Vaishali Muralidaran, Milos D. Ikonomovic, David C. Bloom, Vishwajit L. Nimgaonkar, Leonardo D’Aiuto

**Affiliations:** a Department of Neurology, University of Pittsburgh School of Medicine, Pittsburgh, Pennsylvania, USA; b Geriatric Research Education and Clinical Center, Pittsburgh VA Healthcare System, Pittsburgh, Pennsylvania, USA; c Department of Psychiatry, The Second Xiangya Hospital, Xiangya School of Medicine, Central South University, Changsha, China; d Department of Psychiatry, Western Psychiatric Institute and Clinic, University of Pittsburgh School of Medicine, Pittsburgh, Pennsylvania, USA; e Department of Molecular Genetics & Microbiology, University of Florida College of Medicine, Gainesville, Florida, USA; f VISN 4 MIRECC, VA Pittsburgh Healthcare System, Pittsburgh, Pennsylvania, USA; University of California, Irvine

**Keywords:** Alzheimer's disease, brain organoids, herpes simplex virus 1, HSV-1, induced pluripotent stem cells, iPSCs

## Abstract

Alzheimer's disease is a progressive neurodegenerative disease characterized neuropathologically by the presence of extracellular amyloid plaques composed of fibrillar amyloid beta (Aβ) peptides and intracellular neurofibrillary tangles. Postmortem and *in vivo* studies implicate herpes simplex virus 1 (HSV-1) infection in the brain as a precipitating factor in disease/pathology initiation. HSV-1 infection of two-dimensional (2D) neuronal cultures causes intracellular accumulation of Aβ42 peptide, but these 2D models do not recapitulate the three-dimensional (3D) architecture of brain tissue. We employed human induced pluripotent stem cells (hiPSCs) to compare patterns of Aβ42 accumulation in HSV-1-infected 2D (neuronal monolayers) and 3D neuronal cultures (brain organoids). Akin to prior studies, HSV-1-infected 2D cultures showed Aβ42 immunoreactivity in cells expressing the HSV-1 antigen ICP4 (ICP4^+^). Conversely, accumulation of Aβ42 in ICP4^+^ cells in infected organoids was rarely observed. These results highlight the importance of considering 3D cultures to model host-pathogen interaction.

**IMPORTANCE** The pathogen hypothesis of Alzheimer’s disease (AD) proposes that brain HSV-1 infection is an initial source of amyloid beta (Aβ) peptide-containing amyloid plaque development. Aβ accumulation was reported in HSV-1-infected 2D neuronal cultures and neural stem cell cultures as well as in HSV-1-infected 3D neuronal culture models. The current study extends these findings by showing different patterns of Aβ42 accumulation following HSV-1 infection of 2D compared to 3D neuronal cultures (brain organoids). Specifically, 2D neuronal cultures showed Aβ42 immunoreactivity mainly in HSV-1-infected cells and only rarely in uninfected cells or infected cells exposed to antivirals. Conversely, 3D brain organoids showed accumulation of Aβ42 mainly in noninfected cells surrounding HSV-1-infected cells. We suggest that because brain organoids better recapitulate architectural features of a developing brain than 2D cultures, they are a more suitable model to investigate the involvement of HSV-1 in the onset of AD pathology.

## Abstract (precis)

The pathogen hypothesis of Alzheimer’s disease (AD) proposes that brain HSV-1 infection is an initial source of amyloid beta (Aβ) peptide-containing amyloid plaque development. Aβ accumulation was reported in HSV-1-infected 2D neuronal cultures and neural stem cell cultures as well as in HSV-1-infected 3D neuronal culture models.

## INTRODUCTION

Alzheimer's disease (AD) is a leading cause of dementia, yet its etiology is unknown and there is no effective therapy to prevent or arrest the disease. Primary neuropathological lesions of AD are extracellular plaques of fibrillized amyloid-β (Aβ) peptides and intracellular neurofibrillary tangles of hyperphosphorylated tau (p-tau) protein (1). Aβ plaques develop early in AD and can influence tau phosphorylation (2) and neuroinflammation (3), two processes more closely tied to cognitive impairment (4). This series of events was formalized as the amyloid cascade hypothesis of AD (5). Involvement of infective pathogens in AD pathogenesis was first proposed by Alois Alzheimer (6) and is supported by several recent studies that suggested that Aβ is an antimicrobial peptide (AMP) that plays a role in protecting neurons from infectious pathogens like bacteria and viruses (7, 8). In this regard, increased Aβ production could be a protective response to infection but may lead to excessive Aβ accumulation and, possibly, fibrillization and deposition as amyloid plaques. Supporting this hypothesis, the neurotropic pathogen HSV-1 was detected in Aβ plaques in AD brains (9). *In vitro* studies using two-dimensional (2D) monolayer neuronal cultures reported that HSV-1 infection resulted in intracellular Aβ accumulation and upregulation of β-secretase and the nicastrin component of γ-secretase, which are required for the generation of Aβ from its precursor protein (10). This phenomenon could be due to intermittent cycles of HSV-1 reactivation in the brain (11). A recent study using an *in vitro* three-dimensional (3D) model of human brain also described extracellular accumulation of Aβ42 following infection with HSV-1 (12), possibly due to HSV-1-induced Aβ overproduction/secretion and/or seeding of Aβ aggregation in the extracellular space by virus particles as an antimicrobial response (13). However, not well-understood are the mechanisms underlying altered Aβ production and accumulation during lytic HSV-1 infection or repeated cycles of viral reactivation and how HSV-1-infected cells communicate with nearby cells to initiate this response.

We have recently reported on the use of induced pluripotent stem cells (iPSCs) to model HSV-1 acute and latent infection in 2D monolayer neuronal cultures and 3D brain organoids (14). These *in vitro* models showed that iPSC-derived CNS neurons are permissive for HSV-1 infection, with reporter gene expression being detected from both immediate-early (ICP0) and strict late (gC) promoters and infectious virus being released into the medium during the acute infection period of both 2D and 3D cultures. In addition, we demonstrated that HSV-1 can establish latency in both culture systems. Perhaps the most novel finding of these studies was that 3D brain organoids, but not 2D monolayer neuronal cultures, have the ability to recapitulate the difficulty that HSV-1 has in reactivating from latency in the central nervous system (CNS), as observed in animal models (14). This ability contributes to the potential superiority of 3D brain organoids to model host-pathogen interactions within the CNS.

In this study, we employed iPSC-derived 2D monolayer neuronal cultures and 3D brain organoids to investigate the accumulation of Aβ42 in response to HSV-1 infection. This investigation compared the relative amount of HSV-1-induced Aβ42 in the two models following infection.

## RESULTS

**FIG 1:**
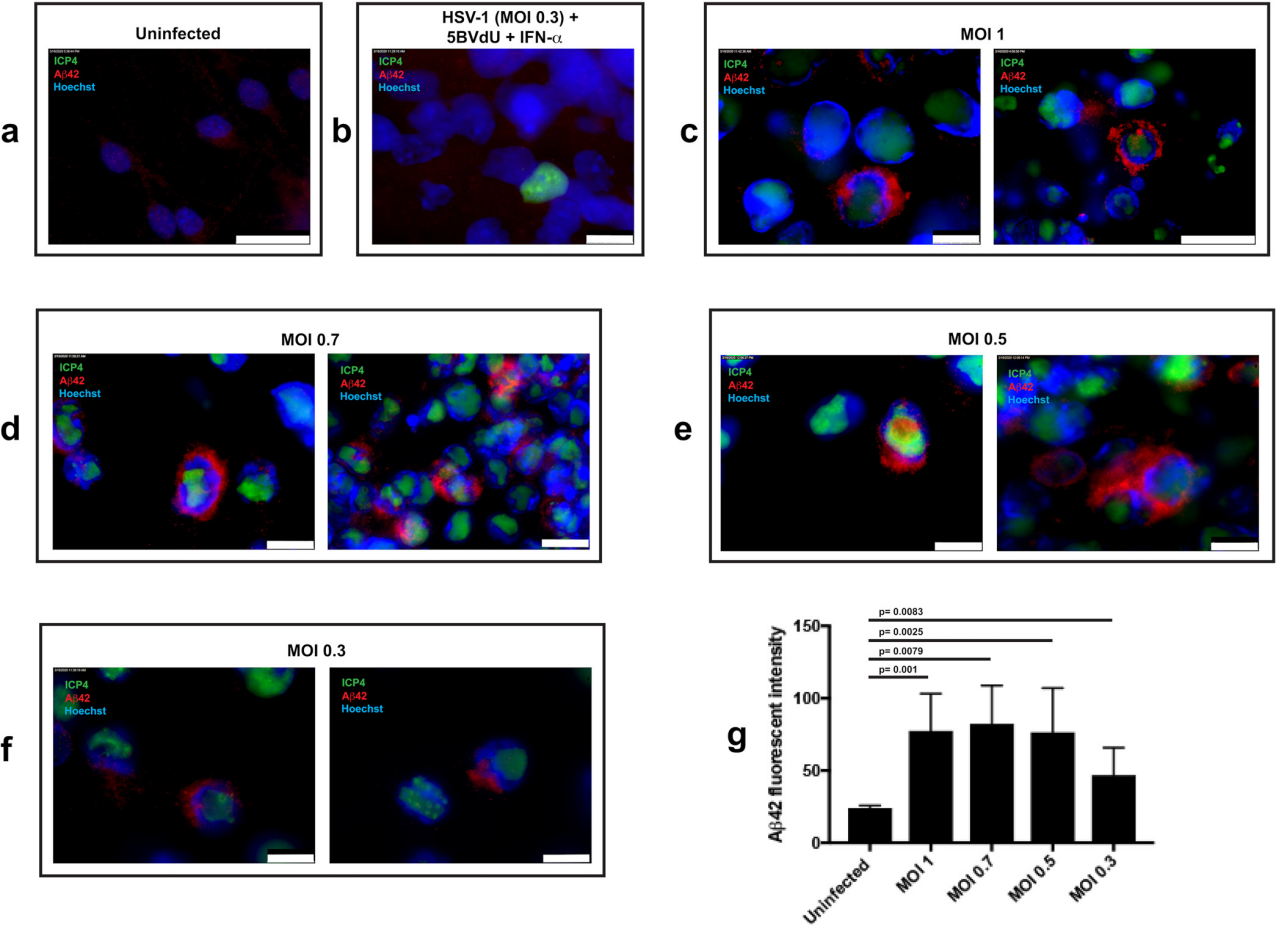
Immunofluorescence for amyloid-β42 and the HSV-1 antigen ICP4 in uninfected and HSV-1-infected hiPSC-derived neurons. hiPSC-derived 2D neuronal cultures were infected at MOIs of 1 to 0.3. Neurons were also infected at an MOI of 0.3 in the presence of antivirals 5BVdU and IFN-α. (a to f) Microphotographs depicting immunostaining of Aβ42 and the HSV-1 antigen ICP4 in uninfected neurons (a), neurons infected at an MOI of 0.3 in the presence of 5BVdU and IFN-α (b), and neurons acutely infected at MOIs of 1 to 0.3 (c to f). Scale bar is 25 μm (a and c, right), 10 μm (b; c, left; d; e; and f). Quantification of Aβ42 immunofluorescence intensity using ImageJ (g). The increase in Aβ42 fluorescence intensity in infected neurons compared to uninfected neurons was assessed using Student's *t* test. Error bars represent standard deviations.

We initially investigated Aβ42 accumulation in 2D neuronal cultures derived from human induced pluripotent stem cells (hiPSCs) and acutely infected at a multiplicity of infection (MOI) of HSV-1, strain KOS, of 1, 0.7, 0.5, and 0.3. To block productive viral replication, neurons infected at an MOI of 0.3 were also maintained in the presence of antivirals [(E)-5-(2-bromovinyl)-2′-deoxyuridine (5BVdU) and interferon alpha (IFN-α)]. The expression of an HSV-1 lytic gene, infected-cell polypeptide 4 (ICP4), and Aβ42 in uninfected and infected cells was analyzed by immunocytochemistry (ICC) at 48 h postinfection (p.i.) (Fig. 1). Neurons in uninfected cultures showed faint, punctate Aβ42 immunoreactivity surrounding the nucleus (Fig. 1a). When cultures were infected at an MOI of 0.3 with the antivirals 5BVdU and IFN-α, no increase in Aβ42 immunoreactivity was observed, even in the rarely observed ICP4^+^ cells (Fig. 1b). When 2D cultures were infected with HSV-1 at MOIs of 1.0, 0.7, and 0.5, intense Aβ42 immunoreactivity was observed in approximately 19% of ICP4^+^ cells but not in any of the uninfected cells in close proximity or distal to infected neurons (Fig. 1c and e). Aβ42 immunoreactivity was observed surrounding and encapsulating the nucleus and consisted of dense puncta and larger globules of more dispersed Aβ42 immunoreactivity. Similar patterns were observed at an MOI of 0.3 in approximately 8% of ICP4^+^ cells, although Aβ42 immunoreactivity was less intense (Fig. 1f to g). These results, in line with other reports (10, 15), show that HSV-1 induces Aβ42 accumulation in infected monolayer cultures of neurons and that antiviral treatment prevents this accumulation during this experimental time frame (Fig. 1b to f).

**FIG 2:**
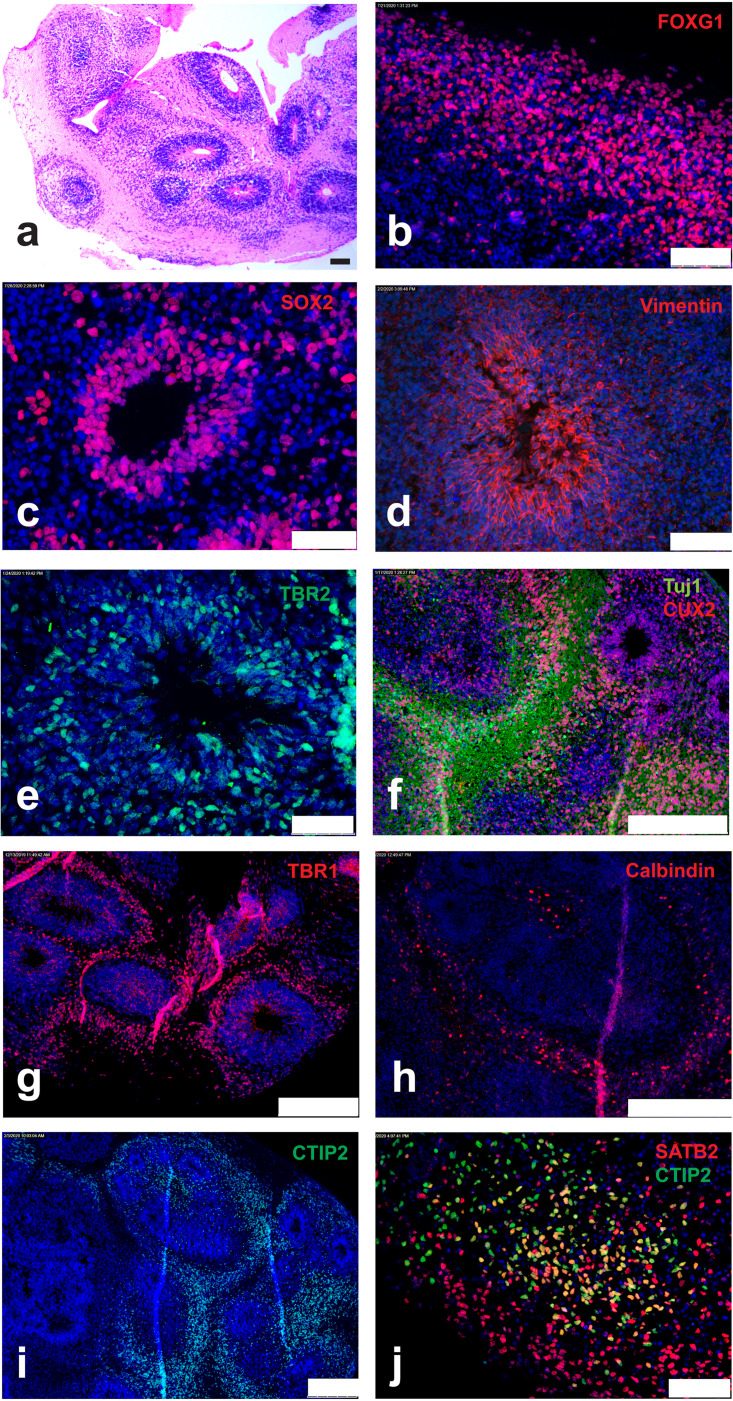
Characterization of 3D brain organoids. (a) Hematoxylin and eosin staining of 9-week-old organoids. (b to j) Immunostaining of 8-μm-thick sections of organoids with antibodies recognizing FOXG1 (b), SOX2 (c), vimentin (d), TBR2 (e), Tuj/CUX2 (f), TBR1 (g), calbindin (h), CTIP2 (i), and CTIP2/SATB2 (j). Nuclei were counterstained with Hoechst 33342. Scale bars are 100 μm (a), 75 μm (b, d, and j), 50 μm (c and e), and 250 μm (f, g, h, and i).

Next, we investigated the suitability of 3D neuronal cultures (i.e., brain cortical organoids) to model HSV-1-induced Aβ accumulation. Figure 2 illustrates the immunohistochemical characterization of a representative noninfected 3D brain organoid at 9 weeks *in vitro*. Cortical brain organoids were generated from hiPSC-derived neural rosettes (for details regarding the differentiation procedure and characterization, see the Materials and Methods).

**FIG 3:**
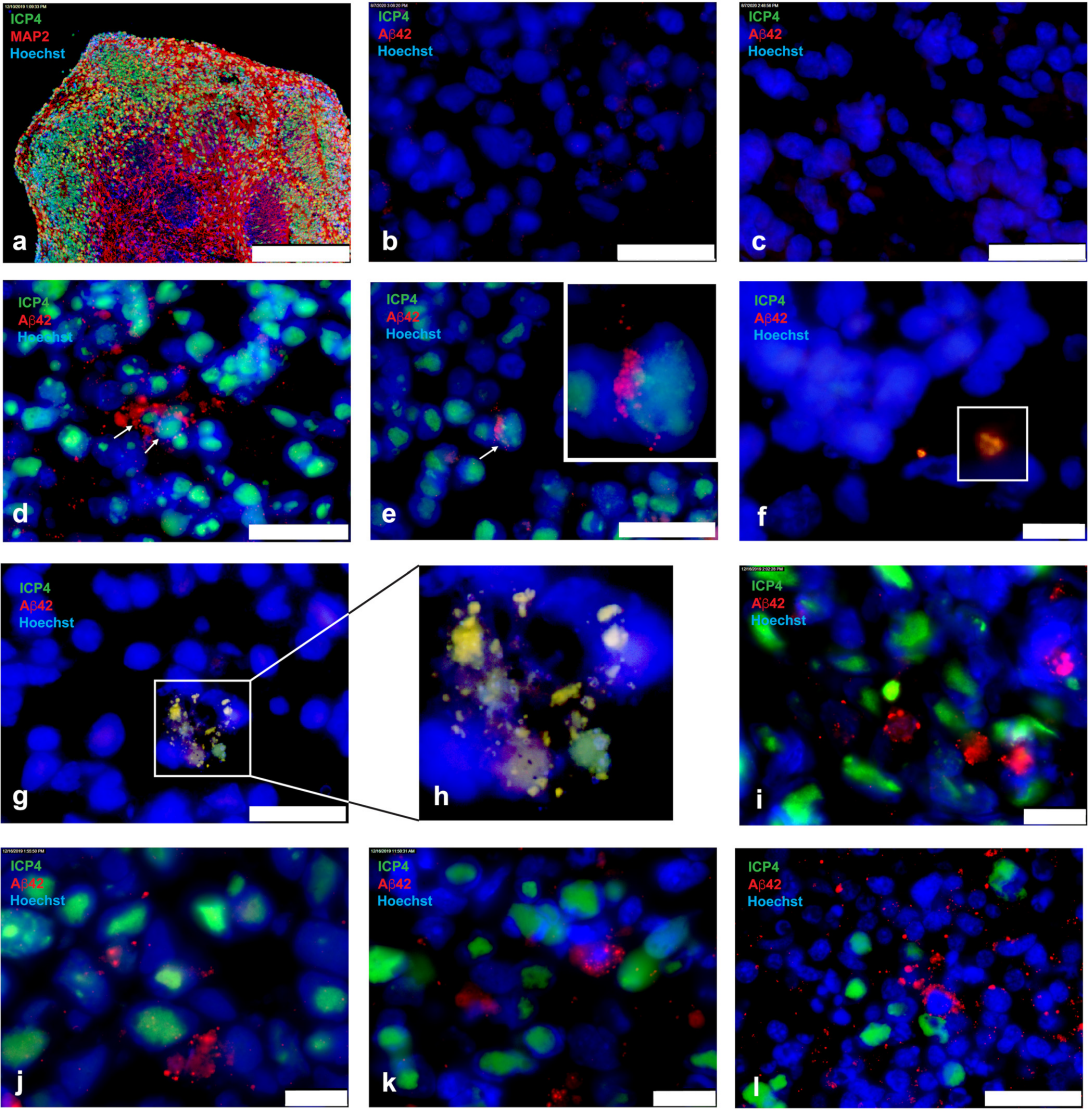
Immunofluorescence for amyloid-β42 and the HSV-1 antigen ICP4 in uninfected and HSV-1-infected 3D brain organoids. Twelve-week-old organoids were infected singularly with 3,000 PFU in the presence or absence of antivirals 5BVdU and IFN-α. After 2 h, the inocula were removed and the organoids were cultured in the presence or absence of antivirals. After 72 h, the organoids were processed for immunofluorescence. (a) Microphotographs of 8-μm-thick sections of HSV-1-infected (a) and uninfected (b) organoids immunostained with ICP4/MAP2 and infected organoids in the presence (c) or absence (d to l) of antivirals with ICP4/Aβ42. Nuclei were counterstained with Hoechst 33342. ICP4^+^ cells exhibiting intracellular Aβ42 immunoreactivity are indicated by arrows. The insets contain enlarged details. Scale bars are 250 μm (a), 25 μm (b, c, d, e, g, and l), and 10 μm (f, i, j, and k).

Twelve-week-old organoids (∼2-mm size) were infected singularly with HSV-1 strain KOS, using 3,000 PFU/organoid. The inocula were removed after 2 h, and organoids were analyzed at days 3 and 5 postinfection. Aβ accumulation was assessed by immunohistochemistry (IHC) analysis of sections obtained from formalin-fixed, paraffin-embedded organoids. IHC showed ICP4^+^ neurons throughout and up to ∼250 μm from the edge of the organoids (Fig. 3a). HSV-1-infected organoids exposed to the antivirals 5BVdU and IFN-α had no detectable ICP4^+^ cells (Fig. 3b), and Aβ42 immunoreactivity was comparable to that of uninfected organoids (Fig. 3c). Contrary to what was observed in infected 2D neuronal cultures, Aβ42 immunoreactivity in ICP4^+^ cells was rarely observed in infected 3D organoids (Fig. 3d and e). In these rare instances, Aβ42 immunoreactivity was observed mainly in areas that appeared to be between infected nuclei (Fig. 3d and e), and perinuclear punctate Aβ42 immunoreactivity was not as robust as that in 2D cultures. In rare instances, Aβ42 immunoreactivity overlapped ICP4^+^ particles (Fig. 3f). Colocalization of ICP4 with large areas of Aβ42 immunoreactivity containing fragmented nuclei also was observed (Fig. 3g and h). Overall, in infected organoids, perinuclear Aβ42 immunoreactivity was predominantly detected in ICP4^−^ cells surrounding ICP4^+^ infected cells (Fig. 3i to l). The robust perinuclear Aβ42 immunoreactivity in ICP4^−^ cells resembled those observed in ICP4^+^ neurons in 2D cultures, and Aβ42 was present between nuclei in extracellular spaces. These observations demonstrate that in the 3D brain organoid model (in contrast to 2D neuronal monolayers), Aβ42 accumulates primarily in ICP4^−^ cells.

## DISCUSSION

The pathogen hypothesis of AD posits that Aβ peptides are produced as a form of antimicrobial protection (AMP) in response to pathogens invading the CNS (10, 16, 17). Specifically, HSV-1 infection could be a trigger for persistent Aβ overproduction and Aβ plaque development (13), either alone or in combination with impaired Aβ clearance. Aβ exhibits antimicrobial properties (18) against a range of bacterial pathogens (19), influenza A virus (20), and herpesviruses (13), and its accumulation in AD may be a side effect of a host innate response to microbial invasion. Among the number of neurotropic viruses that infect the brain, HSV-1 in particular has been proposed as an initial source of Aβ plaque development (13). Recent studies have shown Aβ accumulation in HSV-1-infected 2D neuronal cultures and neural stem cell cultures, which could be interpreted as a mechanism initiated to prevent virus spread (21–24). A recent study provided evidence that Aβ exerts its AMP role by mediating viral agglutination (13). Based on these observations, the following questions emerge: how common is Aβ accumulation in HSV-1 infected cells, and is the 2D cell monolayer model the optimal choice for studying this phenomenon? Our current immunohistochemical analysis of HSV-1-infected 2D monolayer neuronal cultures showed high frequencies of perinuclear Aβ42 immunoreactivity in ICP4^+^ cells compared to uninfected cells or infected cells exposed to antivirals 5BVdU and IFN-α. The frequency of ICP4^+^ cells exhibiting Aβ42 immunoreactivity was progressively lowered by reducing the inoculum to an MOI of 0.3. These results support the ability of HSV-1 to trigger Aβ42 accumulation. However, when we infected 3D brain organoids with HSV-1, Aβ42 immunoreactivity exhibited a pattern distinct from that observed in 2D neuronal monolayers. Specifically, Aβ42 immunoreactivity was observed primarily in ICP4^−^ neurons but rarely in ICP4^+^ neurons. A possible explanation for different patterns of Aβ42 immunoreactivity in 2D monolayers versus 3D brain organoids is that the spread of infection and Aβ42 production occurs at lower rates in the latter model, providing more translational value in studies of brain pathology. It is important to consider the possibility that antiviral factors produced in infected monolayer 2D neuronal cultures are diluted out in the culture medium, while within brain organoids, these factors reach at least the minimal concentration required to exert their antiviral activity. Furthermore, cell-to-cell communication, which is known to be impaired in 2D cultures (25), may contribute to the differences observed between these two *in vitro* systems. It is also important to consider that the ICP4^−^ cells that exhibit an increased Aβ42 immunofluorescence may represent abortively infected cells rather than uninfected cells (26). A recent study by Drayman et al. on sorted cell populations to investigate HSV-1 infection at the single-cell level showed that antiviral response is initiated in ICP4^−^ cells (26). The antiviral genes IFIT1 and IFIT2 are specifically upregulated in the ICP4^−^ cells. In general, interferon-stimulated genes are more highly expressed in cells with low HSV-1 gene expression. Thus, it is possible that the increased Aβ42 immunofluorescence we observed in ICP4-negative cells is the consequence of strong antiviral signaling elicited in this population of abortively infected cells. Though rarely detected in uninfected organoids, it is possible that a fraction of cells expressing Aβ42 were present in organoids prior to HSV-1 infection and were ICP4^−^ due to the peptide’s AMP activity.

In summary, we demonstrate that altered Aβ immunoreactivity due to HSV-1 infection differs in 3D brain organoids from that in 2D neuronal monolayers. In brain organoids, this response is associated with Aβ42 immunoreactivity in ICP4^−^ cells. This differs from 2D monolayer cultures, where HSV-1 infection leads to Aβ42 accumulation primarily in infected ICP4^+^ cells. Because 3D brain organoids of human iPSC recapitulate the 3D architecture of brain, they may be a more suitable model to study the interaction of neuronal HSV-1 infection or repeated cycles of viral reactivation with Aβ alterations in the context of a human neurodegenerative disease. Furthermore, brain organoids may provide critical insights into the mechanisms underlying the communication between HSV-1-infected cells and other cells in the same neuronal network, which can initiate Aβ pathology. Clarifying the role of HSV-1 in AD pathogenesis could lead to antiviral prophylactic or intervention trials, a novel approach supported by a recent report of lower dementia risk associated with anti-herpetic medication for HSV-1 (27).

## MATERIALS AND METHODS

### Cell lines.

Two hiPSC lines, 73-56010-01 SA and 73-56010-02, were employed in this study. Both hiPSC lines were generated from fibroblasts derived from skin biopsy samples that were collected from a healthy volunteer via 4-mm full-thickness punch biopsies under local anesthesia. All identifying information pertaining to the healthy volunteer was removed, and the hiPSCs were established at the National Institute of Mental Health (NIMH) Center for Collaborative Studies of Mental Disorders-funded Rutgers University Cell and DNA Repository (http://www.rucdr.org/mental-health) (RUCDR). All cells were cultured under standard conditions (37°C, 5% CO, and 100% humidity).

### Generation of 2D neuronal cultures.

The neural progenitor cells (NPCs) were derived from iPSCs as previously described (28) and cultured in neurobasal medium supplemented with 2% B27 and 10 ng/ml brain-derived neurotrophic factor (BDNF) for 6 weeks. Half of the culture medium was changed every other day.

### Generation of 3D neuronal cultures (brain organoids).

hiPSCs were cultured in neural progenitor selection medium including dual SMAD inhibitors SB431542 (10 μM) and LDN193189 (100 nM) (NPS/Dual-SMAD medium). After 4 to 5 days in NPS/Dual-SMAD, neural rosettes (250 to 500 μm in diameter) were dissected manually and cultured in NPS/Dual-SMAD medium on ultralow attachment plates for another 2 to 3 days. Neural rosettes were then replated to Matrigel-coated plates. After overnight incubation, the aggregates containing 1 to 4 neural rosettes were isolated manually, transferred into 10-cm petri dishes, and cultured in cortical organoid differentiation medium I (Dulbecco’s modified essential medium [DMEM]-F12–neurobasal [1:1, vol/vol]) supplemented with 1× GlutaMAX, 1× B-27 (VitA[−]), 0.5× nonessential amino acids, 0.5× N-2, insulin (2.5 μg), and 1× penicillin-streptomycin (P/S) on an orbital shaker. One week later, the organoids were transferred into cortical organoid differentiation medium II (DMEM-F12–neurobasal [1:1, vol/vol]) supplemented with 1× GlutaMAX, 1× B-27 (VitA[+]), 0.5× nonessential amino acids, 0.5× N-2, insulin (2.5 μg), BDNF (10 ng/ml), and 1× P/S. Culture medium was changed every 3 days.

Early-stage neocortical organoids (day 33) exhibited a mosaic of ventricular zone (VZ)-like structures most of them containing a single central ventricle-like cavity (Fig. 2a). The forebrain identity of the developing organoids was supported by their expression of FOXG1 (Fig. 2b). Neural progenitor cells in the VZ-like structures expressed sex-determining region Y-box 2 (SOX2) (Fig. 2c). Immunostaining for vimentin (a marker of radial glial cells [29]) in the VZ-like regions was also observed (Fig. 2d). The intermediate progenitor cells in the VZ-subventricular zone (SVZ)-like regions were highlighted by the expression of T-box brain protein 2 (TBR2) (Fig. 2e). Cut-like homeobox 2 (Cux2)-immunoreactive neural precursor cells (Fig. 2f), which are found in the SVZ and the intermediate zone during early stages of brain development and differentiate into upper layer neurons, were observed (Fig. 2f). Neuronal differentiation in the cortical organoids is shown by the expression of the neuronal markers neuron-specific class III beta-tubulin (TUJ1) and microtubule-associated protein 2 (MAP2) (Fig. 2f and 3a). Immunohistochemistry for deep cortical layers markers T-box brain protein 1 (TBR1), calbindin, and chicken ovalbumin upstream promoter transcription factor interacting protein 2 (CTIP2) (Fig. 1g to i), and the superficial layers neurons expressing special AT-rich sequence-binding protein 2 (SATB2) (Fig. 1j) were observed. Overall, these results indicate that the organoids employed in this study exhibit architectural features of a developing brain.

### Viral infections.

HSV-1 strain KOS (VR-1493; ATCC) was employed in this study.

### (i) Infection of 2D neuronal cultures.

For lytic infections, cell-free virus was adsorbed onto monolayer cultures of hiPSC-derived neurons at a range of multiplicities of infection (MOIs), from 1 to 0.3. One hour after the infection, the inocula were removed, and cells were washed twice with DMEM-F12 medium and cultured with neurobasal medium for 48 h. For latent infections, cells were preincubated with 5BVdU plus IFN-α. After 24 h, cells were infected at an MOI of 0.3 and cultured with neurobasal medium in the presence of 5BVdU and IFN-α for 48 h.

### (ii) Infection of brain organoids.

Brain organoids were transferred singularly in 1.5-ml Eppendorf tubes and washed with 500 μl of DMEM-F12 medium. The medium was then discarded, and 50 μl of neurobasal medium with or without 5BVdU and IFN-α containing 3,000 PFU of HSV-1 was added. After perforating the cap of Eppendorf tubes using a 20-gauge sterile needle, the organoids were cultured in an incubator under standard conditions (5% CO_2_, 37°C, and 100% humidity). To inhibit viral replication, the organoids were pretreated with 5BVdU plus IFN-α for 24 h. Two hours after the infection, the inoculum was removed, and the organoids were washed twice with 500 μl of DMEM-F12 medium and cultured singularly in cortical medium II in the presence or absence of 5BVdU and IFN-α in low-attachment 24-well plates on an orbital shaker. Infected organoids were prepared for immunohistochemistry analysis at days 3 and 5 postinfection.

### Immunofluorescence.

The 2D neuronal cultures were fixed with 4% paraformaldehyde and permeabilized with 0.2% Triton-X before immunostaining. The paraffin-embedded slices of organoids were prepared as follows. The organoids were rinsed in phosphate-buffered saline (PBS) and fixed by immersing in at least 10 volumes of 10% formalin overnight at 4°C. The organoids were rinsed again and then embedded in blocks of low-melting-point agarose. The agarose blocks were embedded in paraffin wax by following a standard protocol for formalin-fixed tissue and then sectioned to 5 μm for subsequent staining. Before staining, paraffin sections were incubated at 60°C, dewaxed in xylene, hydrated in absolute, 95%, and 70% ethanol, and rinsed in pure water. Antigen unmasking was performed by exposing paraffin sections to antigen retrieval Citra solution (Biogenex) at 95°C. Paraffin sections were incubated with SuperBlock blocking buffer (Thermo Scientific) before immunostaining. Paraffin sections were also stained with hematoxylin and eosin reagents.

Samples were incubated with primary antibodies overnight at 4°C. Primary antibodies used were mouse monoclonal anti-β-tubulin III antibody (conjugated clone TUJ1; NL1195V; 1:100 dilution; R&D Systems), mouse monoclonal anti-MAP2 antibody (AB5622, 1:500 dilution; Millipore), rabbit polyclonal anti-calbindin antibody (AB11426, 1:400 dilution; Abcam), rabbit polyclonal anti-vimentin antibody (AB45939, 1:500 dilution; Abcam), rabbit polyclonal anti-CUX-2 antibody (AB130395, dilution 1:200; Abcam), mouse monoclonal anti-HSV-1 ICP4 antibody (AB6514, dilution 1:200; Abcam), rabbit polyclonal anti Aβ42 antibody (AB5078P, 1:500 dilution; Millipore), rabbit polyclonal anti-FOXG1 antibody (18259, 1:1,000 dilution; Abcam), rat monoclonal anti-CTIP2 antibody (AB18465, 1:500 dilution; Abcam), rabbit polyclonal anti-TBR1 antibody (AB31940, 1:500 dilution; Abcam), chicken polyclonal anti-TBR2 antibody (AB15894, 1:500 dilution; Millipore), and rabbit polyclonal anti-SATB2 antibody (AB34735, 1:1,000 dilution; Abcam).

The following fluorophore-conjugated secondary antibodies were used to detect bound primary antibodies: Alexa Fluor 488 goat anti-rabbit (1:300 dilution; Thermo Fisher Scientific), Alexa Fluor 488 goat anti-mouse (A-10680, 1:300 dilution; Thermo Fisher Scientific), Alexa Fluor 594 goat anti-rabbit (A-11012, 1:300 dilution; Thermo Fisher Scientific), Alexa Fluor 594 goat anti-mouse secondary antibody (A-11005, 1:300 dilution; Thermo Fisher Scientific), Alexa Fluor 488 goat anti-chicken secondary antibody (A-11039, 1:300 dilution; Thermo Fisher Scientific), and Alexa Fluor 488 goat anti-rat (711-545-152, 1:300 dilution; Jackson ImmunoResearch Labs). A Leica IL MD LED inverted fluorescence microscope was used for image acquisition.
